# Impact of critical illness and withholding of early parenteral nutrition in the pediatric intensive care unit on long-term physical performance of children: a 4-year follow-up of the PEPaNIC randomized controlled trial

**DOI:** 10.1186/s13054-022-04010-3

**Published:** 2022-05-12

**Authors:** Ilse Vanhorebeek, An Jacobs, Liese Mebis, Karolijn Dulfer, Renate Eveleens, Hanna Van Cleemput, Pieter J. Wouters, Ines Verlinden, Koen Joosten, Sascha Verbruggen, Greet Van den Berghe

**Affiliations:** 1 Clinical Division and Laboratory of Intensive Care Medicine, Department of Cellular and Molecular Medicine, KU Leuven, Herestraat 49, 3000 Leuven, Belgium; 2 Intensive Care Unit, Department of Pediatrics and Pediatric Surgery, Erasmus Medical Center, Sophia Children’s Hospital, Rotterdam, The Netherlands

**Keywords:** Critical illness, Children, PICU, Physical function, Strength, Long term

## Abstract

**Background:**

Many critically ill children face long-term developmental impairments. The PEPaNIC trial attributed part of the problems at the level of neurocognitive and emotional/behavioral development to early use of parenteral nutrition (early-PN) in the PICU, as compared with withholding it for 1 week (late-PN). Insight in long-term daily life physical functional capacity after critical illness is limited. Also, whether timing of initiating PN affects long-term physical function of these children remained unknown.

**Methods:**

This preplanned follow-up study of the multicenter PEPaNIC randomized controlled trial subjected 521 former critically ill children (253 early-PN, 268 late-PN) to quantitative physical function tests 4 years after PICU admission in Leuven or Rotterdam, in comparison with 346 age- and sex-matched healthy children. Tests included handgrip strength measurement, timed up-and-go test, 6-min walk test, and evaluation of everyday overall physical activity with an accelerometer. We compared these functional measures for the former critically ill and healthy children and for former critically ill children randomized to late-PN versus early-PN, with multivariable linear or logistic regression analyses adjusting for risk factors.

**Results:**

As compared with healthy children, former critically ill children showed less handgrip strength (*p* < 0.0001), completed the timed up-and-go test more slowly (*p* < 0.0001), walked a shorter distance in 6 min (*p* < 0.0001) during which they experienced a larger drop in peripheral oxygen saturation (*p* ≤ 0.026), showed a lower energy expenditure (*p* ≤ 0.024), performed more light and less moderate physical activity (*p* ≤ 0.047), and walked fewer steps per day (*p* = 0.0074). Late-PN as compared with early-PN did not significantly affect these outcomes.

**Conclusions:**

Four years after PICU admission, former critically ill children showed worse physical performance as compared with healthy children, without impact of timing of supplemental PN in the PICU. This study provides further support for de-implementing the early use of PN in the PICU.

*Trial registration* ClinicalTrials.gov, NCT01536275; registered on February 22, 2012.

**Supplementary Information:**

The online version contains supplementary material available at 10.1186/s13054-022-04010-3.

## Background

Many critically ill children face long-term health problems and impaired physical, neurocognitive, emotional, and behavioral development [1–6]. These sequelae are observed up to years after hospital discharge and can negatively affect health-related quality of life and academic performance [7, 8]. Qualitative scales and questionnaires are valuable tools for documenting developmental impairments in the daily life functioning of the children after critical illness [6–8]. Certain domains have also been extensively documented with the use of objective, quantitative measurements such as anthropometrics to assess growth and a wide range of neurocognitive function tests to evaluate neurocognitive development [4, 5, 9–11]. General assessment of long-term physical functional capacity in daily life after critical illness, however, still remains limited to the use of scales and questionnaires [12–14].

Several pre-admission and disease-related factors may play a role in bringing about the developmental impairments, but also the medical care provided during the pediatric intensive care unit (PICU) stay may contribute, providing leverage points for identifying interventions able to alleviate these poor outcomes [4, 9–11, 15, 16]. One such intervention is avoiding the early use of supplemental parenteral nutrition (PN) in the PICU, as shown in the long-term follow-up of the multicenter randomized controlled PEPaNIC trial. This study had first shown that, as compared with initiating parenteral nutrition (PN) within 24 h after admission to supplement insufficient enteral nutrition (early-PN), withholding of supplemental PN in the first week in the PICU (late-PN) improved short-term PICU outcomes [17]. However, as underfeeding has been related to worse cognitive performance, behavioral problems, and impaired physical development of otherwise healthy children [18–23], there were concerns about potential adverse long-term consequences of the low caloric and macronutrient intake with late-PN for health and physical and neurocognitive development. Therefore, patients were invited for an extensive investigation of these outcomes 2 and 4 years after PICU admission, in comparison with healthy children. As compared with healthy children, former PICU patients showed worse health status, signs of impaired growth, worse neurocognitive functioning, more emotional and behavioral problems, and worse quality of life [8, 10, 11]. Importantly, as compared with early-PN, late-PN did not worsen any of these outcomes and even attenuated the impairments in executive functioning and visual-motor integration and reduced emotional/behavioral problems 2 and/or 4 years later [10, 11], with differential DNA methylation identified as plausible molecular basis of these effects [24, 25]. It remained unclear, however, whether the timing of initiating PN may also affect the patients’ long-term physical function.

In this study, we compared several quantitative measures of physical performance of former PEPaNIC patients 4 years after PICU admission with those of healthy children with a similar age and sex distribution and investigated whether late-PN versus early-PN differentially affected these outcomes.

## Methods

### Study design and participants

The PEPaNIC randomized controlled trial (RCT) enrolled 1440 critically ill children between June 2012 and July 2015 in the participating PICUs (University Hospitals Leuven, Belgium; Erasmus MC Sophia Children’s Hospital, Rotterdam, Netherlands; Stollery Children’s Hospital, Edmonton, Alberta, Canada; ClinicalTrials.gov, NCT01536275) [17]. The PEPaNIC RCT randomly assigned patients 1:1 to early (early-PN) or delayed (late-PN) initiation of supplemental PN when 80% of targeted calories per age and weight categories was not reached with enteral nutrition. In the early-PN group, supplemental PN was initiated within 24 h of PICU admission. In the late-PN group, supplemental PN was withheld in the first week in the PICU (meaning no PN in most children in view of PICU discharge before day 8). When enteral nutrition covered > 80% of calculated targets, supplemental PN was discontinued. After 1 week in the PICU, PN could be administered when necessary to both groups. In both groups, enteral nutrition was equally initiated as soon as possible and intravenous micronutrients were administered until fully enterally fed.

All surviving patients were eligible for a preplanned long-term follow-up 4 years after PICU admission, assessing health status and physical and neurocognitive development via interview, clinical assessments, and questionnaires. During PICU admission of the child, parents or legal guardians had provided consent to contact them for this long-term follow-up testing. A control group of healthy children, comparable to the patients for age and sex distribution, was recruited in parallel with the patients within the same time window and underwent identical assessments for comparison. Apart from unrelated children, healthy siblings and patients’ relatives were included to control as much as possible for genetic, socioeconomic, and environmental background. Healthy children could only participate if they had not been previously admitted to a neonatal ICU or PICU and had not been admitted to hospital with need for an intravenous line for 7 days or more. Additional exclusion criteria included a history of inborn chronic metabolic diseases requiring a specific diet, such as diabetes, and conditions that require home PN, such as short-bowel syndrome. If the burden of coming to the hospital was considered too high, examinations were performed during consented home visits. Assessors of the 4-year outcomes were physicians, physiotherapists, and experienced pediatric psychologists who had not been involved in the management of the patients during PICU stay and who were strictly masked to treatment allocation [11]. Parents and caregivers were not masked while the child was treated in the PICU, and they were not actively informed about the initial PEPaNIC trial results.

Parents, legal guardians, or the child if 18 years or older gave written informed consent according to local regulations. Each center’s institutional review boards approved the study (ML8052, NL49708.078, Pro00038098), which was performed in accordance with the ethical standards laid down in the 1964 Declaration of Helsinki and its amendments. The full study protocol has been published [11, 26]. Health status, anthropometrics, and neurocognitive outcomes have been reported [11]. Clinical assessment of physical functions reported in the present study was only performed for patients enrolled in Leuven or Rotterdam.

### Outcomes

This study quantitatively assessed several measures of physical functional capacity of the participants, including handgrip strength, functional dynamic balance, functional exercise capacity, and overall daily physical activity.

*Handgrip strength* was measured with a Jamar Plus + digital hand dynamometer (Patterson Medical Ltd., Nottinghamshire, UK). Assessments were performed according to standardized testing position and conditions (including allowance of verbal encouragement) as advised by the American Society of Hand Therapists [27]. Average scores (kilograms) of three measurements were recorded and converted to percentage of force predicted for age and sex [28].

In the *“timed up-and-go” test, assessing functional dynamic balance*, the time was measured needed to stand up from an in height adjustable chair (in sitting position, both feet flat on the ground with a 90-degree angle at the knees), walk a 3 m distance, turn around a cone, walk back to the chair, and sit down again. A handheld stopwatch was used to register time in hundredths of a second. The fastest time of three trials was recorded.

The *6-min walk test* was performed according to the American Thoracic Society guidelines [29] as a measure of *functional exercise capacity* and conducted along a straight, flat, corridor. Participants were instructed to walk, not run, as fast as possible for 6 minutes. The researcher offered support and gave time indications. The distance walked in 6 min was recorded to the nearest meter. Heart rate and peripheral oxygen saturation were measured before and after the test, and the corresponding differences were calculated. Changes in oxygen saturation were also noted as categorical variable indicating whether or not saturation dropped with 3% or more, as a measure of exertional oxygen desaturation [30].

Everyday *overall physical activity* was derived from an ActiGraph device (model wGT3X-BT, ActiGraph Corporation, Pensacola, FL), which records acceleration with a triaxial accelerometer and showed the greatest measurement properties for assessing common movement-related outcomes [31]. Participants were asked to wear the ActiGraph at the right hip for 7 consecutive days during waking hours and remove it only for sleeping and water-based activities (e.g., showering, bathing, or swimming). Parents/participants were asked to complete a daily log to record the time the monitor was worn or taken off. ActiGraph devices and logbooks were returned by post. Based on accelerometry output and log sheets, the data were first reduced manually to delete occasional periods of non-wear time. Participants’ ActiGraph data were only considered for evaluation if activity had been registered for at least 5 out of 7 days (of which at least one weekend day) for at least 8 h per day. Raw data extraction based on 60-s epochs was then performed with ActiLife Software 6.13.4 (ActiGraph Corporation). Several outcome parameters were derived, including total energy expenditure (kcal/kg/day and kcal/kg/hour monitored), degree of physical activity energy expenditure (expressed as metabolic equivalent of task (MET), which is the ratio of working metabolic rate relative to resting metabolic rate), activity intensity-based measures (daily time spent sedentary, or in light (< 3.0 METs), moderate (3.0–5.9 METs), vigorous (6.0–8.9 METs), or very vigorous (≥ 9.0 METs) activity (expressed as percentage of worn/monitored time, Additional file 1: Table S1)), number of Freedson bouts (period of at least 10 min in moderate to vigorous activity), number of sedentary bouts (period of at least 10 min in sedentary state), and number of steps (number/day and number/day/hour monitored). All data were averaged over the registered days.

### Statistical analyses

The number of patients enrolled in this follow-up study was determined by the number of patients included in the primary RCT (*n* = 1440) and the fraction of missing data due to any type of loss to follow-up. Therefore, we could not perform an a priori sample size calculation. However, we calculated that a number of 198 participants would be needed per group to demonstrate a 5% difference [32] among healthy and former critically ill children or among early-PN and late-PN groups in handgrip strength predicted for age and sex with 95% certainty and 80% power.

Data are presented as numbers (proportions), medians (interquartile ranges), or beta-estimates (95% confidence intervals (CI)). Univariable comparisons were made with chi-square (Fisher’s exact) or Wilcoxon rank-sum tests, as appropriate. Multivariable linear or logistic regression analyses to investigate outcome differences between groups simultaneously adjusted for risk factors that may affect the studied outcomes, based on clinical practice and literature. To study outcome differences between patients and healthy control children, the risk factors were age, treatment center, sex, race, geographic origin, language, hand preference, history of malignancy, predefined syndrome (Additional file 1: Methods S1), and educational and occupational status of the parents/caregivers (Additional file 1: Methods S2). Multivariable analyses investigating outcome differences between early-PN and late-PN patients additionally adjusted for admission diagnosis, illness severity upon PICU admission (pediatric index of mortality 3 (PIM3) and pediatric logistic organ dysfunction (PeLOD) scores), malnutrition risk (Screening Tool for Risk on Nutritional Status and Growth), and parental smoking behavior before PICU admission. To assess whether patients who were infants (younger than 1 year) at randomization behaved differently from older children, p values for interaction between age-group and randomization were calculated. Finally, we further adjusted the analyses for BMI Z-score to investigate whether nutritional status could affect any association found with the physical outcomes. If necessary, data were square root transformed to obtain a near-normal distribution of the residuals to meet the linear regression model assumptions. Multicollinearity of the covariates was assessed with the use of the variance inflation factor and was generally not a problem [33].

Statistical analyses were performed with JMP^©^Pro16.1.0 (SAS Institute, Inc., Cary, NC). Two-sided *p* values of 0.05 or less were considered to indicate statistical significance. No corrections for multiple comparisons were done, as the studied outcomes are not independent [24, 34].

## Results

**Fig. 1:**
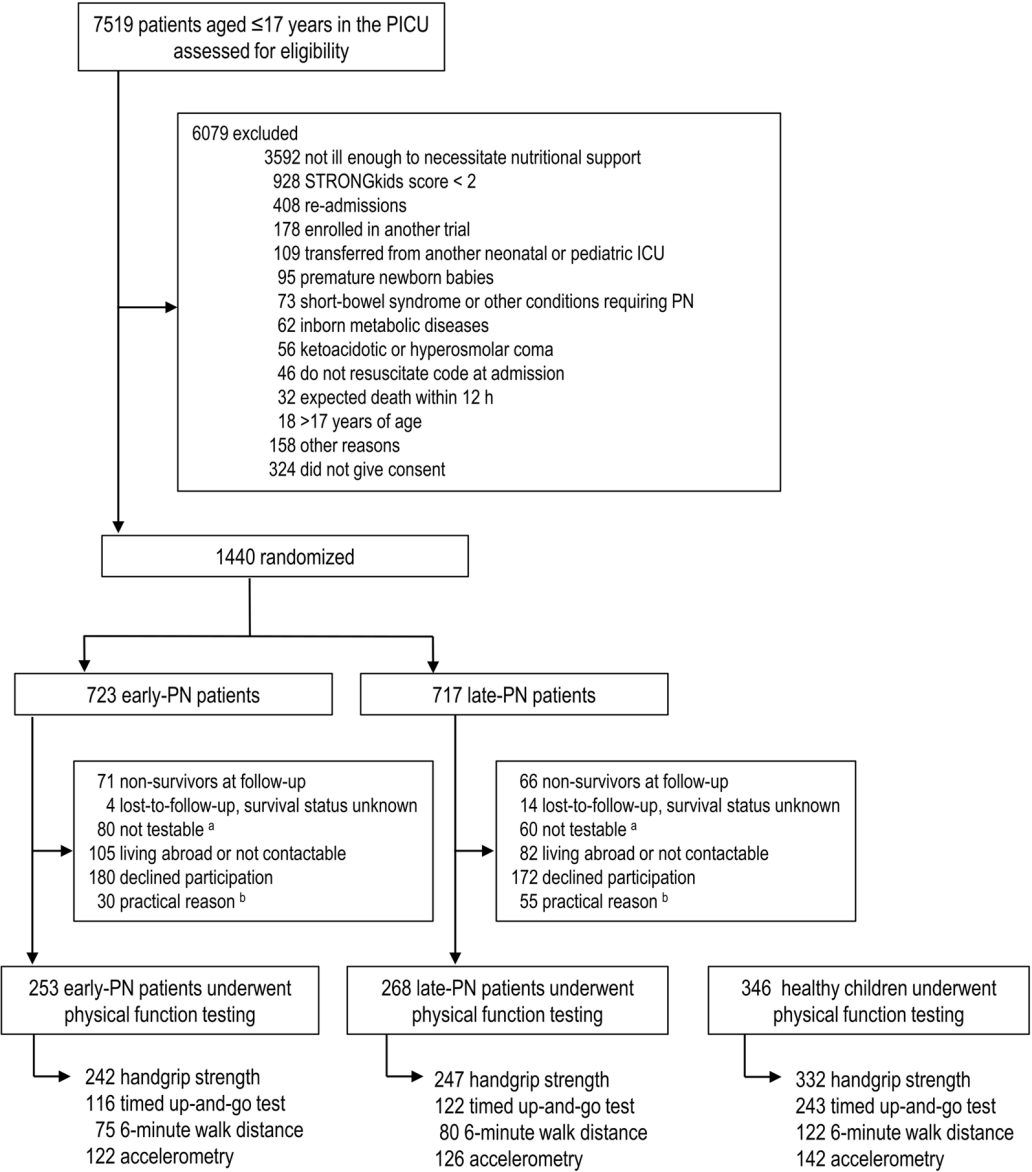
CONSORT diagram of study participants. ^a^These children survived but were physically or neurocognitively disabled (hampering assessment of physical function) or did not understand the instructions. ^b^Practical reasons included, among others, insufficient time (as the physical function testing came last in the follow-up assessments, after a structured interview, anthropometric measurements, a clinical neurological examination, and extensive testing of neurocognitive functions), technical problems, age younger than 4 years, or partial follow-up performed in another center not participating in the PEPaNIC trial. *ICU* intensive care unit, *PN* parenteral nutrition, *STRONGkids* Screening Tool for Risk on Nutritional Status and Growth

Between March 8, 2016, and November 8, 2019, 521 PEPaNIC patients (253 early-PN, 268 late-PN) and 346 healthy children underwent physical function testing (Fig. 1). Demographics and medical characteristics of the participating children are shown in Table 1. Enteral, parenteral, and total macronutrient doses administered on each of the first 7 days of PICU admission for the participating early-PN and late-PN patients are shown in Additional file 1: Fig. S1. Former PICU patients who were tested for physical function (*n* = 521) were comparable for allocation to early-PN or late-PN, for most demographics upon PICU admission, and for PICU primary and secondary study endpoints with those patients who survived, but could not be reached, declined participation in functional testing, or could not be tested due to a practical problem (*n* = 624; Additional file 1: Table S2).

**Table 1:** Participants’ characteristics

Characteristic	Healthy children (*n* = 346)	Patients (*n* = 521)	*p*	Early-PN (*n* = 253)	Late-PN (*n* = 268)
Demographics and anthropometrics					
Age at 4-year follow-up (year), median (IQR)	5.4 (4.4–8.9)	5.1 (4.5–8.5)	0.95	5.1 (4.4–8.4)	5.1 (4.5–8.5)
Male sex, no (%)	184 (53.2)	301 (57.8)	0.18	144 (56.9)	157 (58.6)
Known non-European origin, no (%)^a^	40 (11.6)	78 (15.0)	0.14	44 (17.4)	34 (12.7)
Known non-Caucasian race, no (%)^a^	24 (6.9)	36 (6.9)	0.98	24 (9.5)	12 (4.5)
Known not exclusive Dutch or English language, no (%)	66 (19.1)	110 (21.1)	0.46	51 (20.2)	59 (22.0)
Parental educational level, no (%)^b^			< 0.001		
Educational level 1	11 (3.2)	22 (4.2)		8 (3.2)	14 (5.2)
Educational level 1.5	11 (3.2)	44 (8.5)		27 (10.7)	17 (6.3)
Educational level 2	45 (13.0)	134 (25.7)		66 (26.1)	68 (25.4)
Educational level 2.5	64 (18.5)	96 (18.4)		46 (18.2)	50 (18.7)
Educational level 3	197 (56.9)	147 (28.2)		73 (28.9)	74 (27.6)
Educational level unknown	18 (5.2)	78 (15.0)		33 (13.0)	45 (16.8)
Parental occupational level, no (%)^c^			< 0.001		
Occupational level 1	2 (0.6)	4 (0.8)		0 (0.0)	4 (1.5)
Occupational level 1.5	19 (5.5)	49 (9.4)		19 (7.5)	30 (11.2)
Occupational level 2	40 (11.6)	92 (17.7)		41 (16.2)	51 (19.0)
Occupational level 2.5	24 (6.9)	49 (9.4)		31 (12.3)	18 (6.7)
Occupational level 3	75 (21.7)	101 (19.4)		48 (19.0)	53 (19.8)
Occupational level 3.5	36 (10.4)	37 (7.1)		23 (9.1)	14 (5.2)
Occupational level 4	110 (31.8)	83 (15.9)		36 (14.2)	47 (17.5)
Occupational level unknown	40 (11.6)	106 (20.4)		55 (21.7)	51 (19.0)
Height Z-score at 4-year follow-up (year), median (IQR)	0.37 (− 0.22 to 1.00)	0.17 (− 0.69 to 0.86)	< 0.001	0.27 (− 0.51 to 0.84)	0.12 (− 0.81 to 0.89)
Weight Z-score at 4-year follow-up (year), median (IQR)	0.25 (− 0.23 to 0.88)	0.21 (− 0.54 to 0.86)	0.05	0.20 (− 0.49 to 0.85)	0.24 (− 0.58 to 0.87)
BMI Z-score at 4-year follow-up (year), median (IQR)	0.10 (− 0.56 to 0.69)	0.21 (− 0.48 to 0.95)	0.11	0.16 (− 0.46 to 0.82)	0.21 (− 0.48 to 1.06)
Patient characteristics upon PICU admission					
STRONGkids risk level, no (%)^d^					
Medium	NA	472 (90.6)		28 (11.1)	21 (7.8)
High	NA	49 (9.4)		225 (88.9)	247 (92.2)
PeLOD score first 24 h in PICU, median (IQR)^e^	NA	21 (12–32)		21 (12–31)	22 (12–32)
PIM3 score, median (IQR)^f^	NA	− 3.8 (− 4.4 to − 2.7)		− 3.9 (− 4.5 to − 2.7)	− 3.7 (−4.4 to − 2.8)
PIM3 probability of death (%), median (IQR)^g^	NA	2.2 (1.2–6.1)		2.0 (1.1–6.3)	2.3 (1.2–5.7)
Diagnostic category, no (%)					
Surgery					
Abdominal	NA	42 (8.1)		23 (9.1)	19 (7.1)
Burns	NA	3 (0.6)		2 (0.8)	1 (0.4)
Cardiac	NA	250 (48.0)		117 (46.3)	133 (49.6)
Neurosurgery—traumatic brain injury	NA	41 (7.9)		20 (7.9)	21 (7.8)
Thoracic	NA	30 (5.8)		16 (6.3)	14 (5.2)
Transplantation	NA	7 (1.3)		3 (1.2)	4 (1.5)
Orthopedic surgery—trauma	NA	13 (2.5)		8 (3.2)	5 (1.9)
Other	NA	20 (3.8)		9 (3.6)	11 (4.1)
Medical					
Cardiac	NA	16 (3.1)		8 (3.2)	8 (3.0)
Gastrointestinal–hepatic	NA	1 (0.2)		0 (0.0)	1 (0.4)
Oncologic–hematologic	NA	6 (1.2)		2 (0.8)	4 (1.5)
Neurologic	NA	26 (5.0)		11 (4.4)	15 (5.6)
Renal	NA	0 (0.0)		0 (0.0)	0 (0.0)
Respiratory	NA	43 (8.3)		22 (8.7)	21 (7.8)
Other	NA	23 (4.4)		12 (4.7)	11 (4.1)
History of malignancy, no (%)	0 (0.0)	24 (4.6)	< 0.001	11 (4.4)	13 (4.9)
History of diabetes, no (%)	0 (0.0)	0 (0.0)	> 0.99	0 (0.0)	0 (0.0)
Syndrome, no (%)^h^	2 (0.6)	39 (7.5)	< 0.001	15 (5.9)	24 (9.0)
Known parental smoking between birth and PICU admission, no (%)	NA	136 (26.1)		64 (25.3)	72 (26.9)

Characteristics of former PICU patients and healthy children were comparable, except that the patients’ parental educational and occupational level was lower, and patients suffered more from a history of malignancy or a “syndrome,” as compared with the group of healthy children. Former PICU patients of the early-PN and late-PN group overall had comparable characteristics

*NA* not applicable, *PeLOD* pediatric logistic organ dysfunction score, *PICU* pediatric intensive care unit, *PIM3* pediatric index of mortality 3 score, *STRONGkids* Screening Tool for Risk on Nutritional Status and Growth

^a^Participants were classified according to race and geographical origin by the investigators. These classifications were done to capture the ethnical and regional differences in the frequency of consanguinity, which might adversely affect cognitive performance

^b^The education level is the average of the paternal and maternal educational level and calculated based on the 3-point scale subdivisions as made by the Algemene Directie Statistiek (Belgium) and the Centraal Bureau voor de Statistiek (Netherlands). Low (1), middle (2), and high (3) educational level (Additional file 1: Methods S2)

^c^The occupation level is the average of the paternal and maternal occupation level, which is calculated based on the International ISCO System 4-point scale for professions (Additional file 1: Methods S2)

^d^STRONGkids scores range from 0 to 5, with a score of 0 indicating a low risk of malnutrition, a score of 1 to 3 indicating a medium risk, and a score of 4 to 5 indicating a high risk

^e^PeLOD scores range from 0 to 71, with higher scores indicating more severe illness

^f^Higher PIM3 scores indicate a higher risk of mortality

^g^PIM3 probability of death, ranging from 0 to 100%, with higher percentages indicating a higher probability of death in PICU

^h^A prerandomization syndrome or illness a priori defined as affecting or possibly affecting neurocognitive development (Additional file 1: Methods S1)

### Physical function in former critically ill patients as compared with healthy children

We first compared the performance of former PICU patients with that of healthy children with univariable analyses (Table 2). Former PICU patients had less strength for both hands, needed more time to complete the timed up-and-go test, and were able to walk a shorter distance in 6 min. They showed a more pronounced decrease in peripheral oxygen saturation between the start and end of the walk test, with a higher proportion of children showing a decrease of 3% or more, whereas the corresponding rise in heart rate was comparable to that in healthy children. Registered ActiGraph wear time was slightly shorter in the former PICU patients than in the healthy children. Registered monitoring time revealed a lower total, but not physical activity energy expenditure in former PICU patients. The patients also spent a larger percentage of monitored time performing light activity and a smaller percentage in moderate activity, further underscored by a lower number of Freedson bouts, and walked fewer steps than healthy children. In subsequent multivariable analyses adjusting for risk factors, the differences remained, with no major impact of further adjusting for nutritional status at follow-up (Table 2, Additional file 1: Table S3).

**Table 2:** Physical outcomes at 4-year follow-up of former PEPaNIC patients in comparison with healthy control children

Outcome	Healthy children (*n* = 346)	PEPaNIC patients (*n* = 521)	*p*	Multivariable *p*^c^
Handgrip strength (% of predicted)				
Dominant hand	101.4 (87.0–122.8)	94.0 (77.5–117.0)	< 0.001	< 0.001
Non-dominant hand	99.1 (83.3–120.1)	91.9 (74.3–111.3)	< 0.001	< 0.001
Timed up-and-go test (s)	5.1 (4.5–5.9)	5.5 (4.9–6.3)	< 0.001	< 0.001
6-min walk test				
Distance walked (m)	553 (471–643)	485 (420–587)	< 0.001	< 0.001
Heart rate				
Before the test	88 (74–102)	88 (75–102)	0.62	
After the test	117 (104–129)	120 (103–136))	0.33	
Difference before and after the test	27 (16–40)	29 (16–43)	0.70	0.53
Peripheral O2 saturation				
Before the test	99 (98–100)	99 (97–100)	0.77	
After the test	98 (97–100)	98 (97–99)	0.008	
Difference before and after the test	0 (− 1 to 0)	− 1 (− 2 to 0)	0.04	0.02
3% or more reduced after the test	7 (6.2%)	23 (15.7%)	0.01	0.01
ActiGraph^a^				
Daily time monitored (h)	12.2 (11.6–12.9)	12.0 (11.4–12.7)	0.02	0.02
Total energy expenditure				
kcal/kg/day	121.8 (105.7–182.4)	107.6 (86.6–157.3)	< 0.001	0.007
kcal/kg/hour monitored	10.2 (8.5–14.7)	9.3 (7.2–12.6)	0.001	0.01
Physical activity energy expenditure				
Metabolic equivalent of task (MET)	2.2 (1.7–2.5)	2.1 (1.7–2.4)	0.17	0.26
Daily time spent in a type of activity, %^b^				
Sedentary	46.7 (42.0–54.1)	47.4 (41.5–52.8)	0.65	0.87
Light activity	42.2 (17.7–49.8)	46.1 (21.6–52.2)	0.002	0.001
Moderate activity	8.2 (4.8–26.6)	6.0 (3.9–18.9)	0.01	0.04
Vigorous activity	1.1 (0.5–1.9)	0.9 (0.4–2.0)	0.34	0.31
Very vigorous activity	0.2 (0.0–0.5)	0.1 (0.0–0.6)	0.55	0.97
Moderate to vigorous activity	10.9 (5.7–28.4)	8.0 (4.8–22.5)	0.03	0.08
Number of Freedson bouts	1.0 (0.4–1.8)	0.8 (0.2–1.5)	0.004	0.003
Number of sedentary bouts	6.7 (4.5–9.3)	6.0 (4.4–8.9)	0.29	0.22
Number of steps walked				
Steps/day	9666 (8016–11,109)	8838 (7299–10,308)	0.001	0.007
Steps/day/hour monitored	794 (656–927)	741 (601–867)	0.01	0.05

Data are expressed as median and interquartile range or number and percentage

^a^Data averaged over the registered days

^b^Expressed as the percentage of worn/monitored time

^c^Adjusted for age, treatment center, sex, race, geographic origin, language, hand preference, history of malignancy, a predefined syndrome, and the educational and occupational status of the parents and caregivers

### Physical function in former early-PN and late-PN patients

Patients who had been allocated to late-PN and patients who had been allocated to early-PN during PICU stay performed similarly for handgrip strength, timed up-and-go test, and all aspects of the 6-min walk test, both in univariable and in multivariable analyses (Table 3, Additional file 1: Table S4). Accelerometry output showed a few differences between the groups in univariable analyses, with late-PN patients spending a lower percentage of registered time in moderate or vigorous activity, in accordance with a lower number of Freedson bouts, as compared with early-PN patients. However, no significant differences were found in any of the accelerometry outcomes when adjusting for risk factors in multivariable analyses (Table 3, Additional file 1: Table S4). No interaction was found between age-group (infants versus older children) at the time of PICU admission and randomization to early-PN or late-PN (Additional file 1: Table S5). Further adjustment for nutritional status at follow-up did not change the conclusions (Additional file 1: Tables S4 and S5).

**Table 3:** Physical outcomes at 4-year follow-up of former PEPaNIC patients who had been randomized to early-PN or late-PN

Outcome	Early-PN (*n* = 253)	Late-PN (*n* = 268)	*p*	Multivariable *p*^c^
Handgrip strength force (% of predicted)				
Dominant hand	95.0 (77.4–111.7)	93.6 (77.8–120.3)	0.49	0.27
Non-dominant hand	90.4 (74.5–109.8)	94.4 (73.2–112.9)	0.68	0.59
Timed up-and-go test (s)	5.5 (4.8–6.2)	5.6 (4.9–6.5)	0.27	0.17
6-min walk test				
Distance walked (m)	480 (420–588)	491 (421–582)	0.99	0.67
Heart rate				
Before the test	91 (76–104)	84 (74–101)	0.30	
After the test	117 (102–133)	122 (103–138)	0.33	
Difference before and after the test	26 (13–38)	33 (17–48)	0.12	0.14
Peripheral O2 saturation				
Before the test	99 (96–100)	99 (98–100)	0.11	
After the test	98 (96–99)	98 (97–99)	0.87	
Difference before and after the test	0 (− 2 to 1)	− 1 (− 2 to 0)	0.20	0.11
3% or more reduced after the test	11 (15.5%)	12 (15.8%)	0.96	0.63
ActiGraph^a^				
Daily time monitored (h)	12.0 (11.4–12.7)	11.9 (11.3–12.7)	0.79	0.86
Total energy expenditure				
kcal/kg/day	113.3 (91.4–172.8)	100.9 (85.1–146.3)	0.07	0.30
kcal/kg/hour monitored	9.7 (7.3–13.8)	8.7 (7.0–12.2)	0.10	0.29
Physical activity energy expenditure				
Metabolic equivalent of task (MET)	2.2 (1.8–2.6)	1.9 (1.5–2.3)	0.04	0.16
Daily time spent in a type of activity, %^b^				
Sedentary	47.4 (41.0–53.1)	47.1 (42.3–52.5)	0.53	0.25
Light activity	45.6 (20.8–51.9)	47.3 (34.0–53.3)	0.20	0.24
Moderate activity	7.2 (4.4–22.3)	5.5 (3.7–16.7)	0.04	0.13
Vigorous activity	1.2 (0.5–2.2)	0.7 (0.3–1.7)	0.01	0.17
Very vigorous activity	0.1 (0.0–0.8)	0.1 (0.0–0.5)	0.08	0.07
Moderate to vigorous activity	10.0 (5.8–26.0)	6.5 (4.4–19.3)	0.02	0.09
Number of Freedson bouts	1.0 (0.3–1.7)	0.6 (0.2–1.1)	0.001	0.28
Number of sedentary bouts	6.0 (4.2–9.0)	6.0 (4.7–8.8)	0.57	0.32
Number of steps walked				
Steps/day	9079 (7442–10,831)	8666 (6851–10,060)	0.07	0.38
Steps/day/hour monitored	766 (618–898)	727 (581–834)	0.09	0.43

Data are expressed as median and interquartile range or number and percentage

*PeLOD* pediatric logistic organ dysfunction score, *PICU* pediatric intensive care unit, *PIM3* pediatric index of mortality 3 score, *STRONGkids* Screening Tool for Risk on Nutritional Status and Growth

^a^Data averaged over the registered days

^b^Expressed as the percentage of worn/monitored time

^c^Adjusted for age, treatment center, sex, race, geographic origin, language, hand preference, history of malignancy, a predefined syndrome, the educational and occupational status of the parents and caregivers, admission diagnosis, severity of illness upon PICU admission (PIM3 and PeLOD scores), risk of malnutrition (STRONGkids score), and parental smoking behavior before PICU admission

## Discussion

Children who have been critically ill performed worse for several quantitative measures of physical function 4 years after PICU admission, as compared with matched healthy children. They were weaker in handgrip strength and were slower in completing the timed up-and-go test. They also were able to walk a shorter distance in 6 min and showed a larger drop in peripheral oxygen saturation from before to after this test. Daily life overall physical activity was also affected, as illustrated by a lower energy expenditure, less intense physical activity, and fewer steps walked per day. Timing of initiating supplemental PN in the PICU when enteral nutrition was insufficient did not affect physical performance of the former PICU patients.

Four years after PICU admission, we documented a worse quantitatively measured physical performance and activity of children who have been critically ill as compared with healthy children. This may have wide health implications in view of the importance of adequate physical activity for healthy growth, body composition, cardiorespiratory and musculoskeletal fitness, cardiovascular and metabolic health, motor development, cognitive development, academic achievement, emotional regulation, pro-social behaviors and overall quality of life, during childhood, but also later in life [35, 36], many domains that are known to be affected in former PICU patients. Hence, these data should draw attention to the need of safely stimulating physical activity of these children, considering potential medical limitations and environmental factors [37].

The finding of a decreased physical functional capacity in the long term after PICU discharge is consistent with other studies, with a highly variable post-PICU follow-up time window [12]. However, virtually all these studies used qualitative assessments of general functional status based on a wide variety of questionnaires or scales [12–14, 38]. Only few objective quantitative data are available and this only from small studies (fewer than 50 patients), mostly performed in specific subgroups of former PICU patients. One study did measure handgrip strength of former PICU patients and healthy children, revealing numerically lower strength in the patients, but unfortunately both groups were not comparable for age, and strength was not statistically compared among the groups [38]. Children with severe burn injuries showed lower peak torque and average power output in strength measurements with isokinetic dynamometry 3–4 years later, as compared with healthy children [39]. Also their aerobic capacity, as measured by peak VO_2_ during a standardized treadmill exercise test, and peak heart rate remained lower. Among pediatric patients with acute respiratory distress syndrome, one-third exhibited mild-to-moderate impairments in pulmonary function testing approximately 10 months later, coinciding with a decreased physical functioning quality of life [40]. A significant number of pediatric survivors of acute hypoxemic respiratory failure showed long-term abnormalities in pulmonary function testing, but did not perceive limitations themselves in lifestyle, physical activity, or chronic pulmonary morbidity, although 6-min walk distance appeared lower than predicted [41, 42]. Interestingly, for a shorter distance walked, we observed a more pronounced decrease in peripheral oxygen saturation of former PICU patients in the 6-min walk test as compared with healthy children. The larger decrease translated into a higher odds of exertional oxygen desaturation and was not compensated for by a more pronounced rise in heart rate. Long-term exercise-induced deoxygenation has previously been shown to be highly prevalent in children who survived meningococcal septic shock [43].

Importantly, physical outcome measures were never worse for patients who had been exposed to an early macronutrient deficit in the PICU under the late-PN strategy, as compared with early full feeding of patients under the early-PN strategy. This is reassuring in view of the documented association between malnutrition in children and poorer physical function, such as lower handgrip strength [21–23], and further endorses de-implementation of the use of early-PN in the PICU. The present study also showed that, unfortunately, late-PN also did not improve any measure of physical function, unlike previously demonstrated beneficial effects on several measures of neurocognitive functioning and emotional and behavioral problems [10, 11]. In adult ICU patients, late-PN has been shown to reduce the risk of ICU-acquired muscle weakness and accelerated recovery from such weakness in ICU as compared with early-PN, but long-term impact on physical functional capacity remained unclear [44].

The major strengths of this study include the objective quantitative assessment of physical function measures as opposed to the use of (subjective) qualitative scales and questionnaires, as well as the large sample size of our cohort of critically ill children and own parallel control group of healthy children, as opposed to mostly small studies referring patient data to population norms. This study also has some limitations. First, in the comparison of former critically ill patients with healthy children, we cannot deduce to what extent the impaired physical function in PICU survivors may be explained by preexisting impairments or was evoked by the acute illness or associated treatments in the PICU. However, both previously healthy children with a normal baseline function and those with pre-PICU morbidity and baseline functional impairments have shown a functional decline after PICU discharge, although the capacity for functional recovery may be better in those children with a normal baseline function [45–47]. Second, information on in-hospital physiotherapy or on follow-up consultations and therapies beyond the study protocol were not systematically recorded. Also, no information was available on nutritional status of the participants at 4-year follow-up.

## Conclusions

As compared with matched healthy children, former critically ill children performed worse for several quantitative measures of physical functional capacity 4 years after PICU admission, thus objectifying the adverse physical developmental legacy after pediatric critical illness. Delaying supplemental PN to beyond the first week in the PICU, previously shown to improve short-term outcome and certain long-term neurocognitive and behavioral problems as compared with the early use of PN, did not worsen any of the physical developmental impairments. This study thus provides further support for de-implementing the early use of PN in the PICU.

## Supplementary Information

**Additional file 1.** Compiled file with all Additional information: Additional methods describing the definition of “Syndrome” and the educational and occupational level of the participants’ parents. Additional tables describing cut-off points for intensity of activity measured by ActiGraph; characteristics of PEPaNIC patients who participated in physical function testing in comparison with those who survived but could not be reached, declined participation in functional testing or could not be tested due to a practical problem; multivariable analyses of physical outcomes at 4-year follow-up of former PEPaNIC patients in comparison with healthy control children and of former PEPaNIC patients who had been randomized to early-PN or late-PN, as well as interaction with age-group at randomization. Additional figure describing caloric intake of former critically ill patients during the first 7 days in PICU.

## Data Availability

Data sharing is offered under the format of collaborative projects. Proposals can be directed to the corresponding author.

## Abbreviations

CI: Confidence interval
ICU: Intensive care unit
MET: Metabolic equivalent of task
PeLOD: Pediatric logistic organ dysfunction
PEPaNIC: Early versus late parenteral nutrition in the pediatric intensive care unit
PICU: Pediatric intensive care unit
PIM3: Pediatric index of mortality 3
PN: Parenteral nutrition
RCT: Randomized controlled trial
STRONGkids: Screening Tool for Risk on Nutritional Status and Growth
