# Supplementary material for: Impact of critical illness and withholding of early parenteral nutrition in the pediatric intensive care unit on long-term physical performance of children: a 4-year follow-up of the PEPaNIC randomized controlled trial
Source: Crit Care. 2022 May 12;26:133. doi: 10.1186/s13054-022-04010-3 (PMC9097055; doi:10.1186/s13054-022-04010-3)
Supplement: Supplementary file 1 — Additional file 1. Compiled file with all Additional information: Additional methods describing the definition of “Syndrome” and the educational and occupational level of the participants’ parents. Additional tables describing cut-off points for intensity of activity measured by ActiGraph; characteristics of PEPaNIC patients who participated in physical function testing in comparison with those who survived but could not be reached, declined participation in functional testing or could not be tested due to a practical problem; multivariable analyses of physical outcomes at 4-year follow-up of former PEPaNIC patients in comparison with healthy control children and of former PEPaNIC patients who had been randomized to early-PN or late-PN, as well as interaction with age-group at randomization. Additional figure describing caloric intake of former critically ill patients during the first 7 days in PICU. [file 13054_2022_4010_MOESM1_ESM.docx]

**ADDITIONAL FILE 1**

**ELECTRONIC SUPPLEMENTARY INFORMATION**

**Impact of critical illness and withholding of early parenteral nutrition**

**in the pediatric intensive care unit on long-term physical performance of children:**

**a 4-year follow-up of the PEPaNIC randomized controlled trial**

Ilse Vanhorebeek,^1^ An Jacobs,^1^ Liese Mebis,^1^ Karolijn Dulfer,^2^

Renate Eveleens,^2^ Hanna Van Cleemput,^1^ Pieter J Wouters,^1^ Ines Verlinden,^1^

Koen Joosten,^2^ Sascha Verbruggen,^2^ Greet Van den Berghe^1^

^1^ Clinical Division and Laboratory of Intensive Care Medicine, Department of Cellular and Molecular Medicine, KU Leuven, Leuven, Belgium; ^2^ Intensive Care Unit, Department of Pediatrics and Pediatric Surgery, Erasmus Medical Center, Sophia Children’s Hospital, Rotterdam, The Netherlands

Address correspondence to: Greet Van den Berghe, Clinical Division and Laboratory of Intensive Care Medicine, KU Leuven, Herestraat 49, B-3000 Leuven, Belgium. Phone: 32-16-34-40-21; Fax: 32-16-34-40-15; Email: greet.vandenberghe@kuleuven.be

**TABLE OF CONTENTS**

**Methods S1:** Definition of “Syndrome”

**Methods S2:** Educational and occupational level of the parents

**Table S1:** Cut-off points for intensity of activity measured by Actigraph

**Table S2:** Baseline characteristics and acute effects of randomization for PEPaNIC-patients who participated in physical function testing in comparison with those who survived but could not be reached, declined participation in functional testing or could not be tested due to a practical problem

**Table S3:** Physical outcomes at 4-year follow-up of former PEPaNIC-patients in comparison with healthy control children: multivariable analyses

**Table S4:** Physical outcomes at 4-year follow-up of former PEPaNIC-patients who had been randomized to early-PN or late-PN: multivariable analyses

**Table S5:** Physical outcomes at 4-year follow-up of former PEPaNIC-patients who had been randomized to early-PN or late-PN: interaction with age group at randomization

**Figure S1:** Caloric intake of former critically ill patients during the first 7 days in PICU

**Methods S1: Definition of “Syndrome”**

A pre-randomization syndrome or illness a priori defined as affecting or possibly affecting neurocognitive development, and which is subdivided in the following categories:

- Genetically confirmed syndrome or pathogenic chromosomal abnormality

- Clearly defined syndrome, association or malformation without (identified) genetic aberration

- Polymalformative syndrome of unknown etiology

- Clear auditory or visual impairment without specified syndrome

- Congenital hypothyroidism due to thyroid agenesis

- Brain tumor or tumor with intracranial metastatic disease

- Pediatric psychiatric disorder (e.g. autism spectrum disorder, (treatment for) attention deficit hyperactivity disorder)

- Severe medical disorder, not primarily neurologic, but suspected to alter psychomotor and/or mental performance

- Severe neonatal problem (e.g. severe asphyxia)

- Severe craniocerebral trauma or near-drowning

- Severe infectious encephalitis or drug-induced encephalopathy

- Infectious meningitis, encephalitis or Guillain-Barré

- Resuscitation and/or need for extracorporeal membrane oxygenation prior to randomization

- Severe convulsions or stroke prior to randomization

**Methods S2: Educational and occupational level of the parents**

*Educational level of parents*

The education level is the average of the paternal and maternal educational level, and calculated based upon the 3-point scale subdivisions as made by the Algemene Directie Statistiek (Belgium; statbel.fgov.be/nl/) and the Centraal Bureau voor de Statistiek (The Netherlands; statline.cbs.nl): Low (=1), middle (=2) and high (=3) educational level.

*Occupational level of parents*

The occupation level is the average of the paternal and maternal occupation level, which is calculated based upon the International Isco System 4-point scale for professions (<http://www.ilo.org/public/english/bureau/stat/isco/>). In case one of the parents filled in two jobs in the questionnaire, the highest Isco code level was used. In case “unemployed”, “disabled”, “student”, or “housewife/houseman” was filled in, an Isco code level of 1 was given to that parent. When the parents described their profession as “employee”, “worker”, “liberal profession”, or “retired”, they were given an Isco code level of 2.

**Table S1: Cut-off points for intensity of activity measured by Actigraph**

| **Type of activity** | **Children < 6 years** | **Children 6-18 years** | **Adults** |
| --- | --- | --- | --- |
| Sedentary | 0–819 cpm | 0–149 cpm | Not applicable |
| Light | 820–3907 cpm | 150–499 cpm | 0–2689 cpm |
| Moderate | 3908–6111 cpm | 500–3999 cpm | 2690–6166 cpm |
| Vigorous | 6112 cpm and higher | 4000–7599 cpm | 6167–9642 cpm |
| Very vigorous | Not applicable | 7600 cpm and higher | 9643 cpm and higher |

cpm: counts per minute.

**Table S2: Baseline characteristics and acute effects of randomization for PEPaNIC-patients who participated in physical function testing in comparison with those who survived but could not be reached, declined participation in functional testing or could not be tested due to a practical problem**

| **Characteristic** | **Patients with physical testing**  **(n=521)** | **Patients who survived but could not be reached, declined participation or could not be tested due to a practical problem**  **(n=624)** | **P** |
| --- | --- | --- | --- |
| **Patient characteristics upon PICU-admission** |  |  |  |
| Randomization to late-PN, no (%) | 268 (51.4) | 309 (49.5) | 0.51 |
| Age at PICU-admission (year), median (IQR) | 1.3 (0.2 – 4.8) | 1.6 (0.2 – 7.3) | 0.15 |
| Infant (<1 year) at PICU admission, no (%) | 243 (46.6) | 277 (44.4) | 0.44 |
| Male sex, no (%) | 301 (57.8) | 358 (57.4) | 0.89 |
| STRONGkids risk level, no (%) ^a^ |  |  | 0.45 |
| Medium | 472 (90.6) | 557 (89.3) |  |
| High | 49 (9.4) | 67 (10.7) |  |
| PeLOD score first 24 hours in PICU, mean (SD) ^b^ | 21 (12 – 32) | 13 (11 – 30) | <0.001 |
| PIM3 score, mean (SD) ^c^ | -3.8 (-4.4 – -2.7) | -3.6 (-4.5 – 2.7) | 0.51 |
| PIM3 probability of death (%), mean (SD) ^d^ | 2.2 (1.2 – 6.1) | 2.6 (1.1 – 6.6) | 0.51 |
| Diagnostic category, no (%) |  |  | <0.001 |
| Surgery |  |  |  |
| Abdominal | 42 (8.1) | 64 (10.3) |  |
| Burns | 3 (0.6) | 7 (1.1) |  |
| Cardiac | 250 (48.0) | 212 (34.0) |  |
| Neurosurgery-traumatic brain injury | 41 (7.9) | 50 (8.0) |  |
| Thoracic | 30 (5.8) | 27 (4.3) |  |
| Transplantation | 7 (1.3) | 16 (2.6) |  |
| Orthopedic surgery-trauma | 13 (2.5) | 16 (2.6) |  |
| Other | 20 (3.8) | 23 (3.7) |  |
| Medical |  |  |  |
| Cardiac | 16 (3.1) | 30 (4.8) |  |
| Gastrointestinal-hepatic | 1 (0.2) | 2 (0.3) |  |
| Oncologic-hematologic | 6 (1.2) | 4 (0.6) |  |
| Neurologic | 26 (5.0) | 46 (7.4) |  |
| Renal | 0 (0.0) | 1 (0.2) |  |
| Respiratory | 43 (8.3) | 100 (16.0) |  |
| Other | 23 (4.4) | 26 (4.2) |  |

**Table S2: Baseline characteristics and acute effects of randomization for PEPaNIC-patients who participated in physical function testing in comparison with those who survived but could not be reached, declined participation in functional testing or could not be tested due to a practical problem (continued)**

| **Characteristic** | **Patients with physical testing**  **(n=521)** | **Patients who survived but could not be reached, declined participation or could not be tested due to a practical problem**  **(n=624)** | **P** |
| --- | --- | --- | --- |
| **Patient characteristics upon PICU-admission** |  |  |  |
| History of malignancy, no (%) | 24 (4.6) | 29 (4.7) | 0.97 |
| History of diabetes, no (%) | 0 (0.0) | 2 (0.3) | 0.11 |
| Syndrome, no (%) ^e^ | 39 (7.5) | 89 (14.3) | <0.001 |
| **Acute effects of randomization to early-PN or late-PN** |  |  |  |
| Duration of stay in the PICU (days), mean (SD) | 3 (2 – 7) | 4 (2 – 7) | 0.39 |
| Patients who acquired a new infection in PICU, no (%) | 74 (14.2) | 67 (10.7) | 0.07 |
| Duration of mechanical ventilatory support (days), mean (SD) | 2 (1 – 4) | 2 (1 – 5) | 0.12 |

^a^ STRONGkids scores range from 0 to 5, with a score of 0 indicating a low risk of malnutrition, a score of 1 to 3 indicating a medium risk, and a score of 4 to 5 indicating a high risk. ^b^ PeLOD scores range from 0 to 71, with higher scores indicating more severe illness. ^c^ Higher PIM3 scores indicate a higher risk of mortality. ^d^ PIM3 probability of death, ranging from 0% to 100%, with higher percentages indicating a higher probability of death in PICU. ^e^ A prerandomization syndrome or illness a priori defined as affecting or possibly affecting neurocognitive development (Additional File 1, Methods S1).

STRONGkids: Screening Tool for Risk on Nutritional Status and Growth, PeLOD: pediatric logistic organ dysfunction score, PICU: pediatric intensive care unit, PIM3: pediatric index of mortality 3 score.

**Table S3: Physical outcomes at 4-year follow-up of former PEPaNIC-patients in comparison with healthy control children: multivariable analyses**

| **Outcome** | **β-estimate or odds ratio (95%CI) ^c^** | **P** | **β-estimate or odds ratio (95%CI) ^d^** | **P** |
| --- | --- | --- | --- | --- |
| Handgrip-strength (% of predicted) |  |  |  |  |
| Average force dominant hand | -4.174 (-6.256 – -2.092) | <0.001 | -4.224 (-6.338 – -2.110) | <0.001 |
| Average force non-dominant hand | -4.249 (-6.319 – - 2.179) | <0.001 | -4.328 (-6.426 – - 2.229) | <0.001 |
| Timed up-and-go test (s) | 0.227 (0.129 – 0.325) | <0.001 | 0.233 (0.133 – 0.332) | <0.001 |
| 6-minute walk test |  |  |  |  |
| Distance walked (m) | -23.646 (-34.277 – -13.014) | <0.001 | -25.463 (-36.078 – -14.848) | <0.001 |
| Difference in heart rate before and after the test | -0.840 (-3.507 – 1.826) | 0.53 | -0.499 (-3.259 – 2.261) | 0.72 |
| Difference in peripheral O2 saturation before and after the test | -0.499 (-0.939 – -0.058) | 0.02 | -0.528 (-0.990 – -0.065) | 0.02 |
| Reduction in peripheral O2 saturation before and after the test of 3% or more | 3.899 (1.325 – 11.480) | 0.01 | 4.751 (1.549 – 14.575) | 0.003 |
| Actigraph ^a^ |  |  |  |  |
| Daily time monitored (h) | -0.132 (-0.247 – -0.017) | 0.02 | -0.141 (-0.256 – -0.027) | 0.01 |
| Total energy expenditure |  |  |  |  |
| kcal/kg/day | -0.071 (-0.123 – -0.019) ^e^ | 0.007 | -0.075 (-0.126 – -0.025) ^e^ | 0.003 |
| kcal/kg/hour monitored | -0.033 (-0.061 – -0.006) ^e^ | 0.01 | -0.035 (-0.062 – -0.008) ^e^ | 0.01 |
| Physical activity energy expenditure |  |  |  |  |
| Metabolic Equivalent of Task (MET) | -0.031 (-0.086 – 0.024) | 0.26 | -0.031 (-0.088 – 0.026) | 0.28 |
| Daily time spent in a type of activity, % ^b^ |  |  |  |  |
| Sedentary | -0.076 (-1.016 – 0.864) | 0.87 | -0.060 (-1.002 – 0.882) | 0.90 |
| Light activity | 3.012 (1.186 – 4.838) | 0.001 | 2.514 (0.962 – 4.066) | 0.001 |
| Moderate activity | -0.043 (-0.085 – -0.001) ^e^ | 0.04 | -0.036 (-0.077 – 0.005) ^e^ | 0.08 |
| Vigorous activity | 0.024 (-0.023 – 0.070) ^e^ | 0.31 | 0.030 (-0.016 – 0.076) ^e^ | 0.19 |
| Very vigorous activity | -0.001 (-0.073 – 0.071) ^e^ | 0.97 | 0.004 (-0.069 – 0.078) ^e^ | 0.90 |
| Moderate to vigorous activity | -0.038 (-0.081 – 0.005) ^e^ | 0.08 | -0.030 (-0.072 – 0.011) ^e^ | 0.14 |
| Number of Freedson bouts | -0.057 (-0.095 – -0.018) ^e^ | 0.003 | -0.056 (-0.095 – -0.017) ^e^ | 0.005 |
| Number of sedentary bouts | -0.011 (-0.028 – 0.007) ^e^ | 0.22 | -0.011 (-0.028 – 0.007) ^e^ | 0.22 |
| Number of steps walked |  |  |  |  |
| Steps/day | -354.879 (-613.888 – -95.870) | 0.007 | -339.785 (-600.497 – -79.073) | 0.01 |
| Steps/day/hour monitored | -19.725 (-40.275 – 0.825) | 0.05 | -18.169 (-38.891 – 2.553) | 0.08 |

Data are expressed as β-estimate or odds ratio and 95% confidence interval. ^a^ Data averaged over the registered days. ^b^ Expressed as the percentage of worn/monitored time. ^c^ Expressed as patients versus healthy children and adjusted for age, treatment centre, sex, race, geographic origin, language, hand preference, history of malignancy, a predefined syndrome, and the educational and occupational status of the parents and caregivers. ^d^ As in ^c^, further adjusted for BMI-for-age Z score. ^e^ Data were square root-square root transformed to reach a near normal distribution of the residuals to meet the assumptions for linear regression.

**Table S4: Physical outcomes at 4-year follow-up of former PEPaNIC-patients who had been randomized to early-PN or late-PN: multivariable analyses**

| **Outcome** | **β-estimate or odds ratio (95%CI) ^c^** | **P** | **β-estimate or odds ratio (95%CI) ^d^** | **P** |
| --- | --- | --- | --- | --- |
| Handgrip-strength (% of predicted) |  |  |  |  |
| Average force dominant hand | 1.382 (-1.099 – 3.863) | 0.27 | 1.524 (-1.014 – 4.063) | 0.23 |
| Average force non-dominant hand | 0.673 (-1.839 – 3.186) | 0.59 | 0.808 (-1.754 – 3.371) | 0.53 |
| Timed up-and-go test (s) | 0.095 (-0.044 – 0.233) | 0.17 | 0.096 (-0.046 – 0.238) | 0.18 |
| 6-minute walk test |  |  |  |  |
| Distance walked (m) | 2.872 (-10.570 – 16.313) | 0.67 | 1.856 (-12.049 – 15.762) | 0.79 |
| Difference in heart rate before and after the test | 2.529 (-0.844 – 5.903) | 0.14 | 2.506 (-1.025 – 6.038) | 0.16 |
| Difference in peripheral O2 saturation before and after the test | -0.523 (-1.169 – 0.123) | 0.11 | -0.459 (-1.141 – 0.222) | 0.18 |
| Reduction in peripheral O2 saturation before and after the test of 3% or more | 1.446 (0.321 – 6.525) | 0.63 | 1.989 (0.448 – 8.823) | 0.36 |
| Actigraph ^a^ |  |  |  |  |
| Daily time monitored (h) | -0.011 (-0.134 – 0.112) | 0.86 | -0.009 (-0.133 – 0.116) | 0.89 |
| Total energy expenditure |  |  |  |  |
| kcal/kg/day | -0.028 (-0.081 – 0.025) ^e^ | 0.30 | -0.020 (-0.070 – 0.030) ^e^ | 0.42 |
| kcal/kg/hour monitored | -0.015 (-0.044 – 0.013) ^e^ | 0.29 | -0.012 (-0.038 – 0.015) ^e^ | 0.39 |
| Physical activity energy expenditure |  |  |  |  |
| Metabolic Equivalent of Task (MET) | -0.052 (-0.126 – 0.022) | 0.16 | -0.064 (-0.143 – 0.016) | 0.11 |
| Daily time spent in a type of activity, % ^b^ |  |  |  |  |
| Sedentary | 0.636 (-0.474 – 1.746) | 0.25 | 0.631 (-0.481 – 1.743) | 0.26 |
| Light activity | 1.324 (-0.923 – 3.570) | 0.24 | 1.262 (-0.643 – 3.168) | 0.19 |
| Moderate activity | -0.039 (-0.090 – 0.012) ^e^ | 0.13 | -0.037 (-0.086 – 0.011) ^e^ | 0.13 |
| Vigorous activity | -0.043 (-0.105 – 0.019) ^e^ | 0.17 | -0.036 (-0.098 – 0.026) ^e^ | 0.25 |
| Very vigorous activity | -0.096 (-0.203 – 0.011) ^e^ | 0.07 | -0.078 (-0.194 – 0.038) ^e^ | 0.18 |
| Moderate to vigorous activity | -0.045 (-0.098 – 0.007) ^e^ | 0.09 | -0.043 (-0.093 – 0.007) ^e^ | 0.08 |
| Number of Freedson bouts | -0.025 (-0.072 – 0.021) ^e^ | 0.28 | -0.026 (-0.073 – 0.022) ^e^ | 0.28 |
| Number of sedentary bouts | 0.010 (-0.010 – 0.030) ^e^ | 0.32 | 0.008 (-0.012 – 0.028) ^e^ | 0.42 |
| Number of steps walked |  |  |  |  |
| Steps/day | -129.080 (-422.383 -164.224) | 0.38 | -113.081 (-410.938-184.776) | 0.45 |
| Steps/day/hour monitored | -9.528 (-33.335 – 14.278) | 0.43 | -8.761 (-32.979 – 15.458) | 0.47 |

Data are expressed as β-estimate or odds ratio and 95% confidence interval. ^a^ Data averaged over the registered days. ^b^ Expressed as the percentage of worn/monitored time. ^c^ Expressed as late-PN versus early-PN and adjusted for age, treatment centre, sex, race, geographic origin, language, hand preference, history of malignancy, a predefined syndrome, the educational and occupational status of the parents and caregivers, admission diagnosis, severity of illness upon PICU-admission (PIM3 and PeLOD scores), risk of malnutrition (STRONGkids score), and parental smoking behavior before PICU-admission. ^d^ As in ^c^, further adjusted for BMI-for-age Z score. ^e^ Data were square root-square root transformed to reach a near normal distribution of the residuals to meet the assumptions for linear regression. PeLOD: pediatric logistic organ dysfunction, PICU: pediatric intensive care unit, PIM3: pediatric index of mortality 3, STRONGkids: screening tool for risk on nutritional status and growth.

**Table S5: Physical outcomes at 4-year follow-up of former PEPaNIC-patients who had been randomized to early-PN or late-PN: interaction with age group at randomization**

| **Outcome** | **P** ^a^ | **P** ^b^ |
| --- | --- | --- |
| Handgrip-strength (% of predicted) |  |  |
| Average force dominant hand | 0.65 | 0.56 |
| Average force non-dominant hand | 0.49 | 0.43 |
| Timed up-and-go test (s) | 0.26 | 0.24 |
| 6-minute walk test |  |  |
| Distance walked (m) | 0.58 | 0.59 |
| Difference in heart rate before and after the test | 0.32 | 0.55 |
| Difference in peripheral O2 saturation before and after the test | 0.44 | 0.49 |
| Reduction in peripheral O2 saturation before and after the test of 3% or more | 0.83 | 0.82 |
| Actigraph ^c^ |  |  |
| Daily time monitored (h) | 0.25 | 0.20 |
| Total energy expenditure |  |  |
| kcal/kg/day ^d^ | 0.27 | 0.14 |
| kcal/kg/hour monitored ^d^ | 0.19 | 0.08 |
| Physical activity energy expenditure |  |  |
| Metabolic Equivalent of Task (MET) | NA | NA |
| Daily time spent in a type of activity, % ^e^ |  |  |
| Sedentary | 0.34 | 0.31 |
| Light activity | 0.13 | 0.12 |
| Moderate activity ^d^ | 0.33 | 0.39 |
| Vigorous activity ^d^ | 0.85 | 0.87 |
| Very vigorous activity ^d^ | NA | NA |
| Moderate to vigorous activity ^d^ | 0.33 | 0.41 |
| Number of Freedson bouts ^d^ | 0.28 | 0.24 |
| Number of sedentary bouts ^d^ | 0.37 | 0.34 |
| Number of steps walked |  |  |
| Steps/day | 0.33 | 0.41 |
| Steps/day/hour monitored | 0.26 | 0.29 |

^a^ Adjusted for age, treatment centre, sex, race, geographic origin, language, hand preference, history of malignancy, a predefined syndrome, the educational and occupational status of the parents and caregivers, admission diagnosis, severity of illness upon PICU-admission (PIM3 and PeLOD scores), risk of malnutrition (STRONGkids score), and parental smoking behavior before PICU-admission. ^b^ As in ^a^, further adjusted for BMI-for-age Z score. ^c^ Data averaged over the registered days. ^d^ Data were square root-square root transformed to reach a near normal distribution of the residuals to meet the assumptions for linear regression. ^e^ Expressed as the percentage of worn/monitored time. NA: not applicable (data only available for children 6 years or older).

**Fig. S1 Caloric intake of former critically ill patients during the first 7 days in PICU**

Daily amount of energy provided by the enteral route, the parenteral route or both (total) are shown for the first 7 days of PICU stay. Bars represent the mean and the whiskers represent the standard error of the mean. The blue bars depict the early-PN group, the red bars depict the late-PN group.
